# Knockout Studies Reveal an Important Role of *Plasmodium* Lipoic Acid Protein Ligase A1 for Asexual Blood Stage Parasite Survival

**DOI:** 10.1371/journal.pone.0005510

**Published:** 2009-05-12

**Authors:** Svenja Günther, Kai Matuschewski, Sylke Müller

**Affiliations:** 1 Division of Infection & Immunity and Wellcome Centre for Parasitology, Faculty of Biomedical and Life Sciences, University of Glasgow, Glasgow, United Kingdom; 2 Department of Parasitology, Heidelberg University, School of Medicine, Im Neuenheimer Feld, Heidelberg, Germany; Singapore Immunology Network, Singapore

## Abstract

Lipoic acid (LA) is a dithiol-containing cofactor that is essential for the function of α-keto acid dehydrogenase complexes. LA acts as a reversible acyl group acceptor and ‘swinging arm’ during acyl-coenzyme A formation. The cofactor is post-translationally attached to the acyl-transferase subunits of the multienzyme complexes through the action of octanoyl (lipoyl): *N*-octanoyl (lipoyl) transferase (LipB) or lipoic acid protein ligases (LplA). Remarkably, apicomplexan parasites possess LA biosynthesis as well as scavenging pathways and the two pathways are distributed between mitochondrion and a vestigial organelle, the apicoplast. The apicoplast-specific LipB is dispensable for parasite growth due to functional redundancy of the parasite's lipoic acid/octanoic acid ligases/transferases. In this study, we show that *LplA1* plays a pivotal role during the development of the erythrocytic stages of the malaria parasite. Gene disruptions in the human malaria parasite *P. falciparum* consistently were unsuccessful while in the rodent malaria model parasite *P. berghei* the *LplA1* gene locus was targeted by knock-in and knockout constructs. However, the *LplA1^(−)^* mutant could not be cloned suggesting a critical role of LplA1 for asexual parasite growth *in vitro* and *in vivo*. These experimental genetics data suggest that lipoylation during expansion in red blood cells largely occurs through salvage from the host erythrocytes and subsequent ligation of LA to the target proteins of the malaria parasite.

## Introduction

Lipoic acid (6,8-thioctic acid, LA) is an essential cofactor for the multienzyme complexes pyruvate dehydrogenase (PDH), α-ketoglutarate dehydrogenase (KGDH), branched chain α-keto acid dehydrogenase (BCDH) and the glycine cleavage system (GCS). These enzyme complexes are composed of three or four subunits which themselves are oligomers [1], [2]. The three subunits that comprise the α-keto acid dehydrogenase complexes (KADH) are the α-keto acid decarboxylase (or E1), the acyltransferase (or E2), which uses lipoamide as a cofactor, and the dihydrolipoamide dehydrogenase (or E3). LA is post-translationally attached to E2 and functions as the so-called “swinging arm” in the reaction catalysed by KADH complexes, accepting the acyl-moiety from E1 and transferring it to coenzyme A to form acyl-CoA [1]. During this reaction the lipoamide cofactor is reduced and subsequently re-oxidised by E3 to regenerate its ability to accept the next acyl-moiety from E1. The GCS works in a similar way to the KADH, with the H-protein being the lipoylated heart of this complex [3].

Usually these enzyme complexes are found in the mitochondrion of eukaryotic cells, but plastid-bearing organisms also possess a PDH in the plastid that provides substrates for type II fatty acid biosynthesis operating in the organelle [4]. This situation requires lipoylation machineries in both the mitochondrion and plastids [5].

In most microorganisms, lipoylation occurs through salvage and subsequent ligation of LA to the target protein employing lipoic acid protein ligases (LplA) [6]. LplA attach scavenged LA to the substrate protein in an ATP-dependent reaction. The first step of the reaction leads to the formation of a LA-AMP intermediate and the release of pyrophosphate. The activated lipoyl-moiety is then attached to the ε-amino group of a specific lysine residue in the lipoyl-domain of the E2-subunit or the H-protein while AMP is released [6]. In the absence of exogenous LA, bacteria synthesise LA *de novo*. They ligate the octanoyl-moiety of octanoyl-acyl carrier protein (ACP), an intermediate of fatty acid biosynthesis, to the E2-apo-proteins or apo-H-protein using octanoyl (lipoyl) : *N*-octanoyl (lipoyl) transferase (LipB). Subsequently, lipoic acid synthase (LipA) catalyses the insertion of two sulphurs into position C6 and C8 of the octanoyl-moiety to form the lipoyl-arm required for KADH and GCS activities [6].

In the apicomplexan parasites *Plasmodium falciparum*, the causative agent of human malaria, and *Toxoplasma gondii*, causing toxoplasmosis, LA metabolism has some peculiar features that might be exploitable for future drug development. The parasites possess a single mitochondrion and a single remnant plastid called apicoplast, and both organelles contain enzyme complexes that require lipoylation [7]–[9]. Consistent with this is that both organelles possess lipoylation machineries [8], [10]–[12]. In contrast to other plastid-containing eukaryotes, the apicomplexan mitochondrion exclusively uses salvaged LA while the plastid cannot salvage LA from its environment but relies solely on *de novo* biosynthesis of the cofactor [13]–[15]. Interestingly, *Plasmodium* possesses two functional LplA-like proteins with LplA1 being present in the mitochondrion and LplA2 showing dual localisation to the mitochondrion and apicoplast [11], [16]. This suggests potential redundancy between the lipoylation pathways and indeed knockout of the apicoplast targeted *LipB* gene was not lethal for *P. falciparum* intraerythrocytic stages suggesting that the dually targeted LplA2 compensates for the loss of LipB function [16]. Down-regulation of acyl carrier protein (ACP) expression in *T. gondii* revealed that lipoylation levels of the apicoplast PDH was ablated suggesting that *de novo* fatty acid biosynthesis operating in the organelle is the major source for the LA precursor octanoyl-ACP [15]. This is similar to a study by Witkowski and colleagues [17] who found that one of the major products of mitochondrial fatty acid biosynthesis in mammalian cells is octanoyl-ACP, which is efficiently ligated to the mitochondrial H-protein. Similarly, it was reported that one of the main roles for mitochondrial fatty acid biosynthesis in plants and the protozoan parasite *Trypanosoma brucei* is the generation of octanoyl-ACP to serve as substrate for LA biosynthesis [18], [19]. In the light of these studies, it appears imperative to obtain further insights into the importance of the mitochondrial LA salvage pathways present in *Plasmodium* which clearly is essential to provide LA to the KADH and GCS with the absence of LA biosynthesis in the mitochondrion. This study aimed to elucidate the role of LplA1 in both *P. falciparum* and *P. berghei* using reverse genetics approaches.

## Results

### Knockout studies of LplA1 in *P. falciparum*

**Figure 1:**
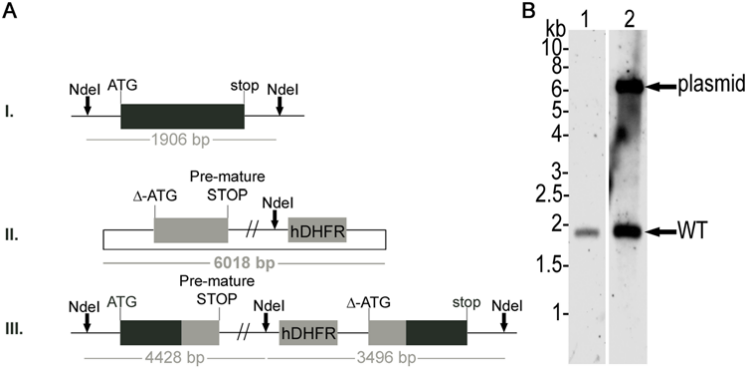
The P. falciparum LplA1 locus cannot be disrupted. *A*. Schematic representation of endogenous *PfLplA1* gene locus (I.), transfection plasmid pHH1-*LplA*-KO (II.) and *PfLplA1* gene locus after single cross-over recombination (III.). The restriction enzyme used for diagnostic digest is shown (*Nde*I) and the expected sizes of diagnostic bands after hybridization with *PfLplA1* or *hDHFR* (human dihydrofolate reductase) probes are indicated. *B*. Southern blot analyses of *P. falciparum* transfected with pHH1-*LplA1*-KO. Genomic DNA of wild-type D10 (lane 1) and *PfLplA1*-KO after 3 selection cycles with WR99210 (lane 2) was digested with *Nde*I and the Southern blot was probed with the *P. falciparum LplA1* ORF. The 1.9 kb band that corresponds to endogenous *PfLplA1* is visible in both lanes, whereas the transfection plasmid pHH1-*LplA1*-KO (6 kb band) only is recognised by the probe in the transfectants. No other bands are visible that are diagnostic for the integration of the *PfLplA1* gene locus.

In order to obtain insights into the importance of LA salvage by LplA1 we decided to attempt to knockout the *LplA1* gene in *P. falciparum*. We first targeted the *P. falciparum LplA1* gene by single cross-over recombination using the construct pHH1-*LplA1*-KO. The KO-construct lacks the ATG start codon and is truncated at the 3′ end so that upon recombination of the plasmid by single cross-over two non-functional gene copies are generated. The first copy retains the endogenous promoter and the start codon but is truncated at the 3′end through the integration of a premature stop codon into the construct and the second copy is without promoter and start codon (Fig. 1A). Two independently transfected parasite lines were generated and each was taken through three drug selection cycles in order to select for parasites where the *PfLplA1* locus had been disrupted by the transfection plasmid. Genomic DNA was isolated and analysed by diagnostic Southern blotting (Fig. 1B). This revealed the presence of endogenous *PfLplA1* (1.9 kb band) and linearised plasmid (6 kb band), but no integration events into the parasite genome were detected (Fig. 1B).

**Figure 2:**
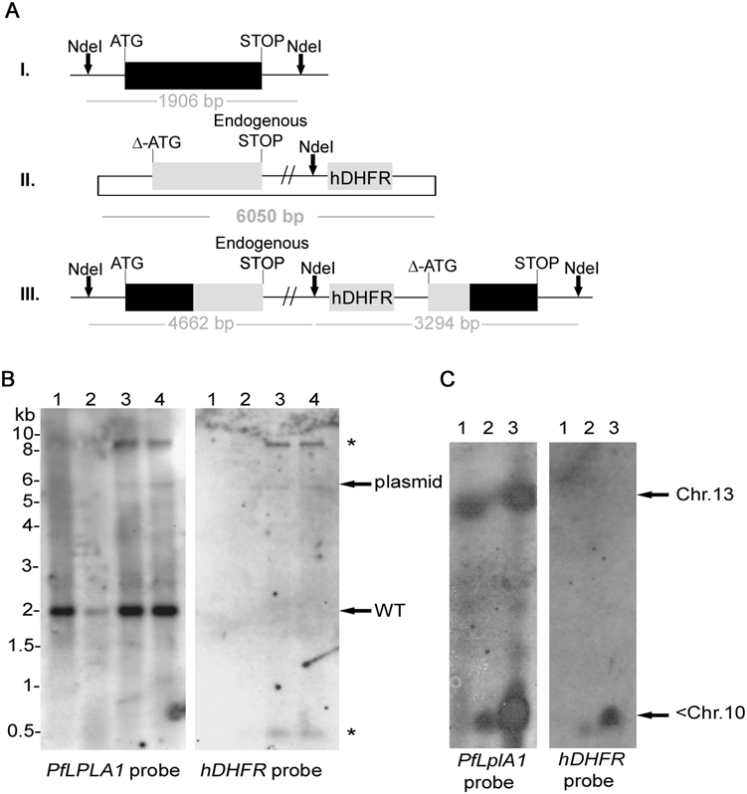
Genotypic analysis of P. falciparum transfected with the complementation construct pHH1-LplA1-KOkon. *A*. Schematic representation of endogenous *PfLplA1* gene locus (I.), transfection plasmid pHH1-*LplA1*-KOkon (II.) and *PfLplA1* gene locus after single cross-over recombination (III.). The restriction enzyme used for diagnostic digest is shown (*Nde*I) and the expected sizes of diagnostic bands after hybridization with *PfLplA1* or *hDHFR* are indicated. *B*. Southern blot analyses of transfected parasite lines. Genomic DNA of wild-type and *LplA1*-KOkon parasites was digested with *Nde*I and probed with the *PfLplA1* ORF (left panel). In all parasite lines analysed, the endogenous gene is present (1.9 kb band) and in lanes 3 and 4 two additional DNA fragments are detected by the probe (in lane 2 less DNA was loaded on the gel so that the plasmid band is hardly visible). The faint 6 kb band is diagnostic for the presence of the transfection plasmid (see scheme), but the band of ∼9 kb (*) cannot be assigned to any specific integration event. Lane 1, wild-type D10; lane 2, *PfLplA*-KOkon, cycle 1; lane 3, *PfLplA*-KOkon, cycle 2; lane 4, *PfLplA*-KOkon, cycle 3. The same blot was stripped and re-probed with the probe specifically detecting the selectable marker *hDHFR* and apart from faint plasmid bands at 6 kb in lanes 2, 3 and 4 two additional bands of ∼9 kb (*) and 0.5 kb (*) are detected. Lane 1, wild-type D10; lane 2, *PfLplA*-KOkon, cycle 1; lane 3, *PfLplA*-KOkon, cycle 2; lane 4, *PfLplA*-KOkon, cycle 3. *C.* Genotyping by pulsed field gel electrophoreses. Chromosomes of wild-type *P. falciparum* D10 (lane 1) and parasites transfected with the pHH1-*LplA1*-KOkon construct in cycle 0 (lane 2) and cycle 3 (lane 3) were analysed by PFGE and the blots were probed with the *PfLplA1* open reading frame (left panel) or with the *hDHFR* open reading frame (right panel) present in the transfection plasmid. The *PfLplA1* probe detected the endogenous *PfLplA1* gene locus on chromosome 13 in wild-type and transfected parasites. However, the probe also detected a strong signal at the bottom of the blot where the non-separated chromosomes <10 are running. The *hDHFR* probe similarly generated signals on chromosome <10, but there is no sign of integration into the *LplA1* gene locus on chromosome 13.

In order to analyse whether this lack of recombination was due to the essential role of *Pf*LplA1 for parasite survival or whether other reasons prevent targeting of the *PfLplA1* gene locus, two approaches were taken. First, parasites were transfected with the knock-in construct pHH1-*LplA1*-KOkon, which retains the entire 3′end. Upon single cross-over recombination the recombinant locus is predicted to contain one functional gene copy whereas the second copy lacks the ATG start codon and should, therefore, be non-functional (Fig. 2A). Similar as described above, parasites were taken through three drug selection cycles after transfection and their genotypes were analysed by Southern blotting (Fig. 2B). Probing the blot with the *PfLplA1* open reading frame (ORF) revealed that after transfection the parasites contained the plasmid (6 kb band) and the endogenous *PfLplA1* gene (1.9 kb band). During drug selection an additional band of ∼9 kb appeared which was also detected by the *hDHFR* probe (Fig. 2B, right panel). This suggests that the pHH1-*LplA1*-KOkon plasmid carrying the selectable marker had targeted a gene locus other than *PfLplA1* since the 9 kb band in the Southern blot did not correspond to the expected diagnostic restriction pattern after recombination with the *PfLplA1* gene locus. It is unknown which gene locus was targeted by the transfection plasmid but some of the regulatory elements present in pHH1-*LplA1*-KOkon potentially might lead to recombination with the parasite genome. The suggestion of a stable integration of pHH1-*LplA1*-KOkon into an unrelated gene locus is not only consistent with the appearance of the prominent ∼9 kb band that cross-reacted with both the *PfLplA1* probe and the *hDHFR* probe but also the concomitant loss of the episome from the parasites despite drug selection. Presumably this stable integration into the parasite genome makes particularly difficult to select for a gene knockout especially if disruption of the gene would result in a growth defect of the null mutants. Similarly, the knock-in of a control plasmid could possibly result in a growth phenotype given that the gene locus is altered by the integration of the transfection plasmid. Confirmation that an unrelated gene locus was targeted by pHH1-*LplA1*-KOkon came from pulsed field gel electrophoresis (PFGE), where the *PfLplA1* probe detected a signal on chromosome 13, which is presumably the endogenous *PfLplA1* gene in wild-type parasites. In contrast, in the *LplA1*-KOkon parasites the probe detected an additional signal that is likely to correspond to the transfection plasmid integrated into an unrelated gene locus present on one of the smaller chromosomes (Fig. 2C; left panel, <Chr.10). The same signal was detected with the *hDHFR* probe corroborating the conclusion that the transfected control plasmid readily targeted a gene locus other than *PfLplA1* (Fig. 2C, right panel).

**Figure 3:**
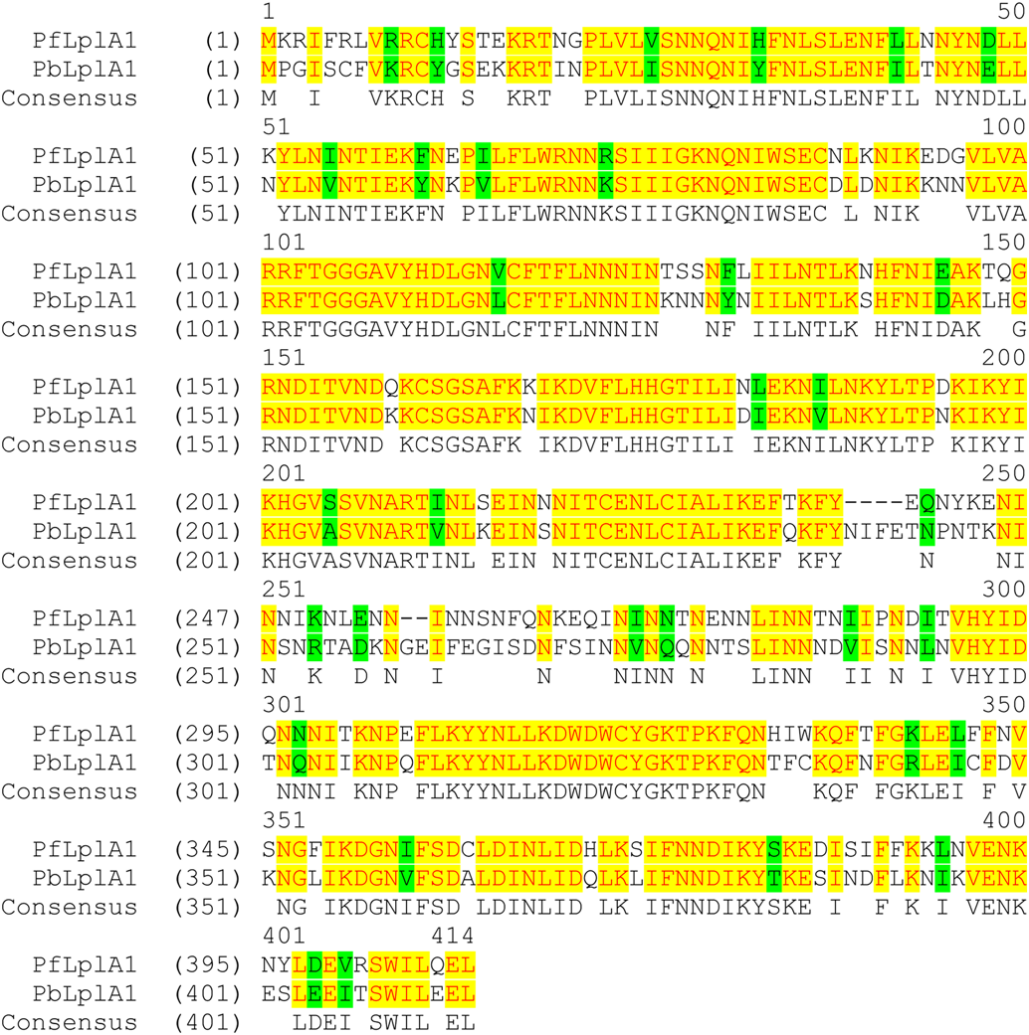
Alignment of *P. falciparum* LplA1 and *P. berghei* LplA1. The deduced amino acid sequences of PF13_0083 and PB 000283.02.0 were aligned using ClustalW. The identity between the two sequences was determined to be 70.3% while the similarity is 78%. The predicted sizes of the two proteins are almost identical with 47.94 kDa for PfLplA1 and 47.97 kDa for *Pb*LplA1. Identical residues are labelled in yellow; homologous residues are labelled in green. Consensus: gives the consensus amino acid sequence of both proteins.

**Figure 4:**
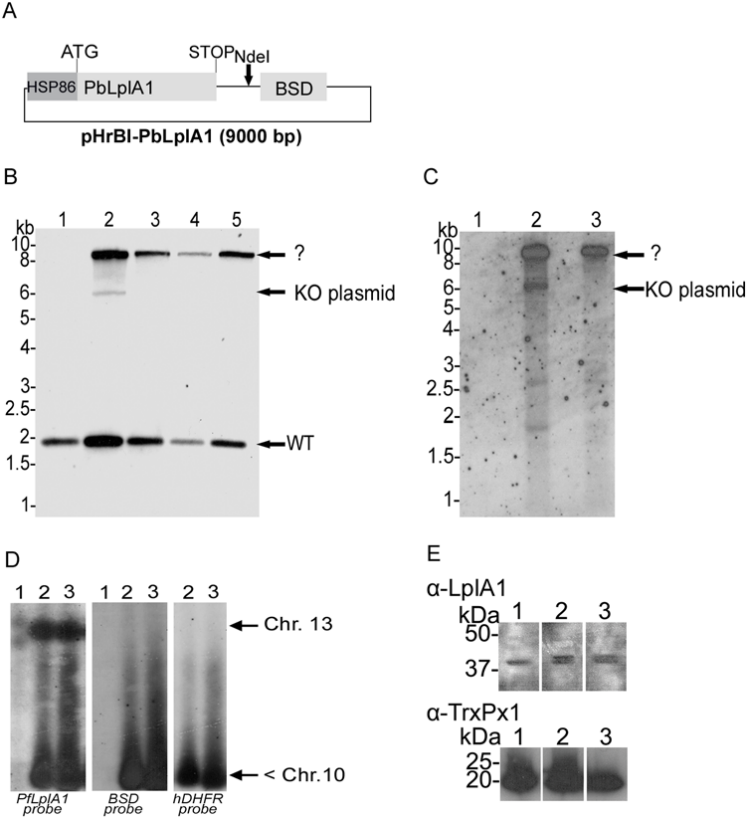
Genotyping of the double transfection in *P. falciparum* with the targeting construct pHH1-*LplA1*-KO and the trans-species expression construct pHrBI-*PbLplA1*. The expression plasmid was transfected into the *P. falciparum* line already carrying pHH1-*LplA1*-KO in cycle 0. *A*. Schematic representation of the plasmid pHrBI-*PbLplA1* carrying the expression cassette of *P. berghei LplA1* which is under the control of the *PfHSP86* promoter. *B.* Genotyping of co-transfected parasite lines. Genomic DNA of wild-type (lane 1) and co-transfected parasites isolated after selection cycle 0 to 3 following transfection (lanes 2 to 5) was digested with *Nde*I, and the blot was probed with the *PfLplA1* open reading frame. The blot showed the endogenous *PfLplA1* gene specific band at 1.9 kb in lane 2 (cycle 0 after transfection). The pHH1-*LplA1*-KO plasmid (6 kb) was visible in cycle 0 (lane 2) and an additional DNA fragment was recognised by the probe, which is unrelated to any expected fragments. The *P. falciparum LplA1* probe did not recognise the presence of the 9 kb pHrBI-*PbLplA1* expression plasmid (see Supplementary Figure 2) but the signal on the blot is due to recombination of pHH1-*LplA1*-KO into an un-related gene locus. The 6 kb pHH1-*LplA1*-KO plasmid band disappears in lanes 3 to 5 while the ∼9 kb band is prominent in these parasite lines. *C.* Using a *hDHFR* probe it was established that the 9 kb band contains both *P. falciparum LplA1* and the selectable marker suggesting that the plasmid had targeted a gene locus unrelated to *PfLplA1*. *D.* Analyses of the co-transfected parasite lines by PFGE supports that the pHH1-*LplA1*-KO plasmid had recombined with an unrelated gene locus (probe *hDHFR*, right panel). Reprobing the blot with the *BSD* probe (middle panel) that specifically recognises the pHrBI-*PbLplA1* expression construct, showed that the expression plasmid was also present on a chromosome that was not resovled under the conditions of this PFGE. The *PfLplA1* locus was recognised by the *PfLplA1*-specific probe (left panel). Lanes 1, wild-type; lanes 2, *P. falciparum* co-transfected with *LplA1*-KO and pHrBI-*PbLplA1*, cycle 0; lanes 3, *P. falciparum* co-transfected with *LplA1*-KO and pHrBI-*PbLplA1*, cycle 3. *E*. Expression of LplA1 protein in co-transfected parasites. The western blot shows parasite extracts that were isolated from wild-type (lane 1) and two independent *P. falciparum* lines co-transfected with pHH1-*LplA1*-KO and pHrBI-*PbLplA1* (lanes 2 and 3) probed with a rabbit anti-*P. falciparum* LplA1 antibody at 1∶1000 dilution. The antibody detects one band in the wild-type parasite extracts that corresponds to the endogenous LplA1 protein. In the co-transfected parasite lines an additional protein is detected, which presumably corresponds to the *P. berghei* LplA1 protein expressed from pHrBI-*PbLplA1*. The blot was re-probed with a rabbit antibody raised against *P. falciparum* 1-Cys peroxiredoxin as a loading control.

**Figure 5:**
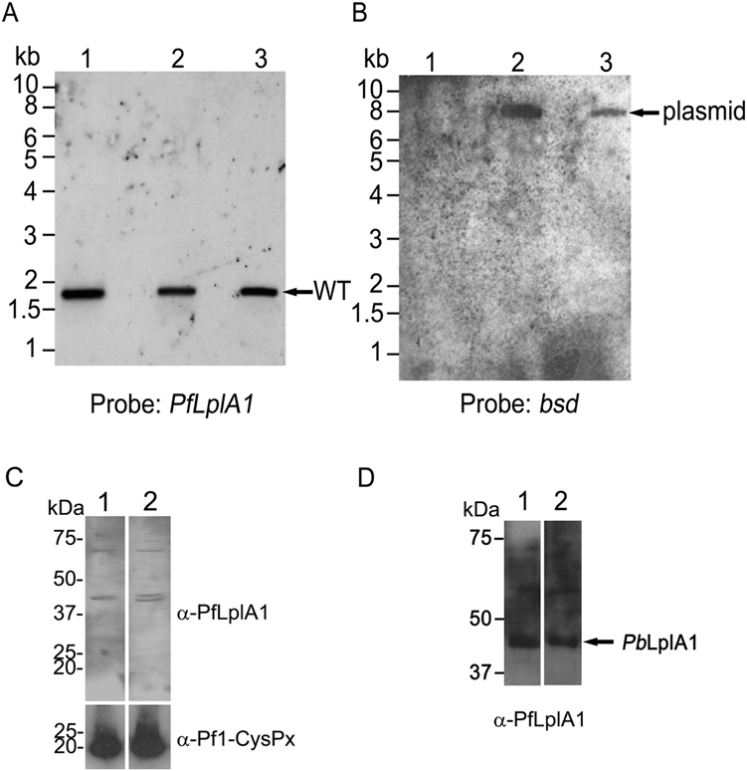
Analysis of *P. falciparum* harbouring pHrBI-*PbLplA1*. *A*. Southern blot of *P. falciparum* transfected with pHrBI-*PbLplA1* before transfection (lane 1) and after transfection (lanes 2 and 3). The DNA was digested with *Nde*I and the blot was probed with the *PfLplA1* ORF. The DNA fragment recognised by the probe corresponds to the endogenous *PfLplA1* gene. The *PfLplA1* probe did not cross-react with the transfected plasmid pHrBI-*PbLplA1*. *B*. To verify the presence of the transfected plasmid, the blot was re-probed with a probe specifically recognising the blasticidin S deaminase ORF present on the plasmid as selectable marker. The probe hybridised with a DNA fragment diagnostic for the transfected plasmid (∼9.0 kb). *C*. Western blot of *P. falciparum* expressing PbLplA1. Lane 1, non-transfected parasites; Lane 2, *P. falciparum* transfected with pHrBI-*PbLplA1*. Equal loading was verified by re-probing the blot with a polyclonal antibody directed against the 1-Cys peroxiredoxin of *P. falciparum*. The polyclonal antiserum raised against *Pf*LplA1 recognised a protein of ∼45 kDa in the wild-type parasites which corresponds roughly to the expected size of *Pf*LplA1 (predicted size 47.9 kDa). In the transfected parasite line carrying the pHrBI-*PbLplA1* plasmid two proteins of very similar size were detected by the antibody – presumably one corresponds to the endogenous *Pf*LplA1 while the second band corresponds to *P. berghei* LplA1. This is surprising as the predicted sizes of both proteins are virtually identical. However, it is possible that the cleavage sites of the mitochondrial targeting peptides are distinct generating proteins that are just distinguishable by SDS-PAGE. *D*. Western blot of *P. berghei* lysate (lane 1: 1×10^6^ parasites and lane 2: 0.2×10^7^ parasites) using *P. falciparum* rabbit anti-LplA1 polyclonal antibody. A band of ∼45 kDa (similar size to the protein identified in the double transfected parasites as well as the wild-type parasites only transfected with pHrBI-*PbLplA1*) reacts strongly with the heterologous antiserum.

As a second approach, parasites that already contained the pHH1-*LplA1*-KO plasmid were transfected with an additional plasmid carrying an expression cassette of *P. berghei LplA1* ORF. It is likely that the *P. berghei* protein can replace the *P. falciparum* LplA1 function given the high degree of amino acid similarity (∼70%) of the two proteins (Fig. 3). Expression of the related *P. berghei LplA1* in pHrBI-*PbLplA1* is controlled by the *P. falciparum* Hsp86 promoter resulting in expression of *P. berghei LplA1* throughout blood stage development [20]. This approach was taken to allow a knockout of the *P. falciparum* gene through pHH1-*LplA1*-KO and concomitantly express a copy of *PbLplA1* from an episome, which should not recombine with the endogenous gene locus. Parasites already bearing the pHH1-*LplA1*-KO episomally were co-transfected with pHrBI-*PbLplA1* (Fig. 4A) and the genotype of the obtained transfectants was analysed (Fig. 4). After transfection (in cycle 0) the endogenous gene was present and in addition two bands of 6 kb and 9 kb, respectively were detected in the blot using the *PfLplA1* probe (Fig. 4 B, lane 2). The 6 kb fragment corresponds to pHH1-*LlpA1*-KO while it is not clear what the 9 kb fragment represents. The possibility that this fragment is due to cross-reactivity between the *PfLplA1* probe and the pHrBI-*PbLplA1* plasmid was ruled out. In a control experiment, where wild-type *P. falciparum* had been transfected with pHrBI-*PbLplA1*, the plasmid was only detected with the *BSD*-specific probe and not the *PfLplA1* probe (Fig. 5A,B). In the following selection cycles, the 6 kb band diagnostic for the KO-plasmid disappeared and the only band in addition to the *PfLplA1* wild-type signal that reacted with both the *PfLplA1* and the *hDHFR* probes was a ∼9 kb fragment (Fig. 4B,C). This suggests that pHH1-*LpA1*-KO had recombined with a gene locus other than *PfLplA1* despite the presence of the episome bearing the *P. berghei LplA1* gene. To further analyse the genotype of the double transfected parasites, PFGE was performed and the blot was probed with the *PfLplA1*-probe, the *hDHFR*-probe and the *BSD*-probe. This experiment revealed that the *PfLplA1* gene was undisrupted on chromosome 13 and that the two plasmids appeared to be present on a chromosome smaller than chromosome 10, which was not resolved at the bottom of the PFGE gel (Fig. 4D). Further, it should be noted that the *PfLplA1* probe also detected the presence of a *LplA1* gene copy on the smaller chromosome suggesting that at least pHH1-*LplA1*-KO still carried the *LplA1-KO* cassette. This suggests that the *BSD*-positive signal on the smaller chromosome also corresponds to the entire pHrBI-*PbLplA1* plasmid. It cannot be excluded that pHH1-*LplA1*-KO and pHrBI-*PbLplA1* had actually recombined with each other before they recombined with the non-related gene locus. This possibility was not further analysed. In order to analyse whether the *PbLplA1* copy that was introduced into the parasites was actually expressed and thus potentially would compensate for *Pf*LplA1 function, western blots were performed. The results show that parasites co-transfected with knockout and expression plasmid express two proteins that are recognised by the *P. falciparum* LplA1 antiserum. The two proteins are 70.3% identical (Fig. 3) suggesting that it is likely that the antibody raised against the *P. falciparum* LplA1 protein cross-reacted with the *P. berghei* protein. This was corroborated by performing a western blot performed on *P. berghei* blood stage parasite lysate (Fig. 5D). Consistent with this, the blot of the double transfectants revealed the presence of two protein bands of about 45 kDa mass that were barely separated from each other while the antibody only recognised a single protein of similar size in the wild-type parasites (Fig. 4E). A similar result was obtained when a *P. falciparum* line that was only transfected with pHrBI-*PbLplA1* was analysed by western blotting, which also revealed that the antiserum raised against *Pf*LplA1 detected two proteins of the expected sizes in these independent transfectants (Fig. 5C). That both proteins appear to migrate faster in the SDS-PAGE than their predicted sizes is likely to be due to the fact that the mitochondrial targeting peptide has been cleaved and the observed sizes correspond to those of the mature proteins. From these experiments we concluded that it is not easily possible to target the *P. falciparum LplA1* gene locus and we tentatively suggest that *LplA1* has an important, if not essential function for *P. falciparum* during their erythrocytic development.

### Knockout studies of LplA1 in *P. berghei*

To further investigate the role of LplA1, knockout of the *LplA1* gene was attempted in the murine malaria parasite *P. berghei*. Two knockout strategies were employed - the first one using single cross-over recombination, similar to the strategy described above for *P. falciparum*, and the second using double cross-over recombination techniques [21].

**Figure 6:**
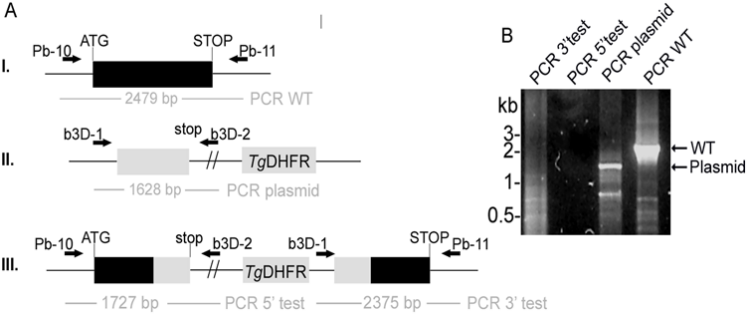
*PbLplA1* is essential for propagation of asexual stages. *A.* Insertion strategy to generate *LplA1* null mutants. The *PbLplA1* genomic locus (I.) is targeted with a *Hpa*I-linearized targeting vector (b3D-LplA1-int1, II.) containing 5′ and 3′ truncations of the *LplA1* open reading frame and the *T. gondii DHFR/TS* positive selectable marker. Upon a single cross-over event, the region of homology is duplicated, resulting in two truncated, non-expressed *LplA1* copies in the recombinant locus (III.). Wild-type, plasmid, and integration-specific primer combinations are indicated by arrows and sizes of expected fragments are shown. *B.* Genotyping indicates absence of successful integration. While episomal plasmid (primer pair b3D-1 and b3D-2 amplifying a 1.63 kb pair; lane 3) and the endogenous *LplA1* gene (primer pair Pb-10 and Pb-11 amplifying a 2.48 kb fragment; lane 4) are amplified, no integration-specific bands (lane 1 and 2: 3′- and 5′-specific integrations, respectively) using the primer pairs b3D-1 and Pb-11 to amplify a 2.38 kb 3′-fragment and Pb-10 and b3D-2 to amplify a 2.45 kb 5′-fragment were obtained from each of 3 transfected parasite populations.

**Figure 7:**
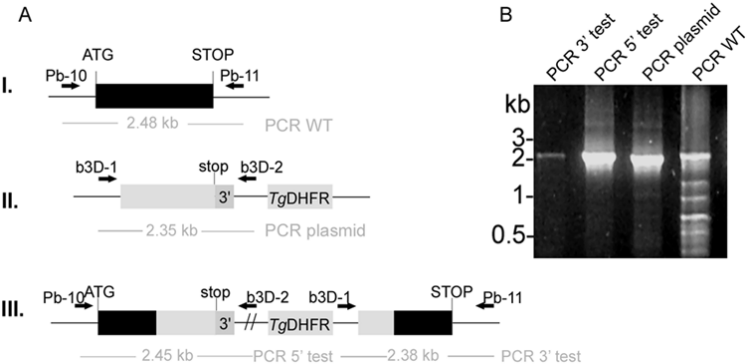
*PbLplA1* is susceptible to gene targeting. *A.* Control integration strategy to recover one functional *LplA1* gene copy. The targeting vector (b3D-LplA1-int2, II.) contains the endogenous stop codon and 3′ untranslated regions of the endogenous *PbLplA1* but lacks the promoter and the start codon. Upon a single cross-over event, one functional *LplA1* copy that is driven by the endogenous promoter and a 5′ truncated copy of the gene are generated (III.). Wild-type, plasmid, and integration-specific primer combinations are indicated by arrows and sizes of expected fragments are shown. *B.* Genotyping indicates successful integration of the control targeting construct. Both integration-specific PCRs (lane 1, 3′ specific integration using primer pair b3D-1 and Pb-11 and lane 2, 5′ specific integration using primer pair Pb-10 and b3D-2) amplified fragments of the expected sizes and their authenticity was verified by nucleotide sequencing. The population still contains episomal plasmid (lane 3; primer pair b3D-1 and b3D-2 amplifying a 2.35 kb band which was subcloned and sequenced) and residual wild-type parasites (lane 4; primer pair Pb-10 and Pb11 fragment was amplified and subcloned and sequenced).

The first strategy should result in a disruption of the endogenous gene locus and generate two incomplete copies of *PbLplA1* - one truncated at the 3′end and one truncated at the 5′end (Fig. 6A). This strategy was also used to generate a knock-in construct which should lead to a reconstitution of the endogenous gene upon integration of the plasmid into the *PbLplA1* gene locus. Parasites were transfected three times with linearised KO-plasmid and after pyrimethamine selection in mice the resistant parasites were subjected to diagnostic PCR analyses (Fig. 6B). According to the PCR results, only endogenous *PbLplA1* and plasmid were amplified (lanes 3 and 4 in Fig. 6B), but no PCR product was obtained with the diagnostic primer sets for a disruption of the *PbLplA1* gene locus (lanes 1 and 2 in Fig. 6B). These data suggest, similar to those described above for *P. falciparum*, that the *LplA1* locus in *P. berghei* might either be refractory to recombination or that *LplA1* knockout cannot be achieved because of the essential function of the gene/protein. The first conclusion can be excluded because transfection of *P. berghei* with the control plasmid b3D-*LplA*-int2 resulted in pyrimethamine resistant parasites that showed integration of the plasmid into the *PbLplA1* gene locus (Fig. 7). The authenticity of the PCR products was verified by sequence analysis confirming that the *LplA1* locus in *P. berghei* is not refractory to recombination.

**Figure 8:**
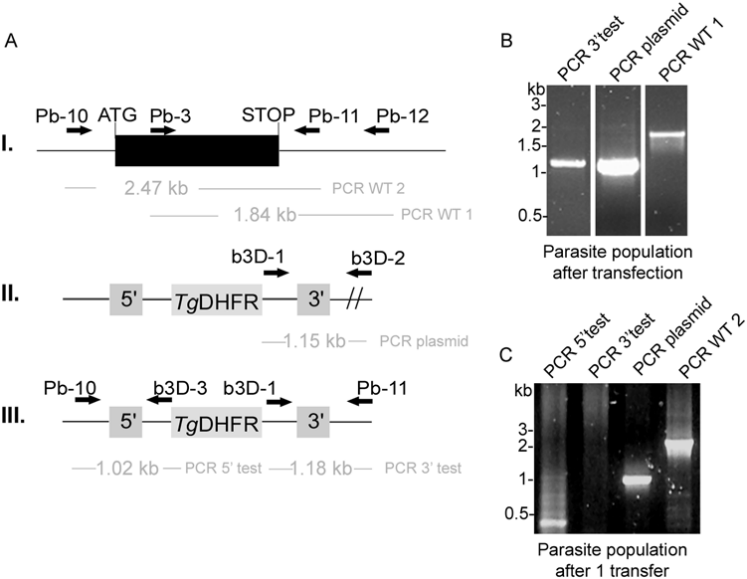
*PbLplA1* is sublethal for asexual parasite growth. *A.* Gene replacement strategy to generate potential *LplA1-REP* null mutants. The wild-type *PbLplA1* genomic locus (I.) is targeted with a *Kpn*I/*Sac*II-linearized replacement vector (b3D-LplA1-REP; II.) containing 5′ and 3′ regions of the *LplA1* open reading frame that flank the *T. gondii DHFR/TS* positive selectable marker. Upon a double cross-over event, the endogenous *LplA1* gene is replaced by the selection marker (III.). Wild-type, plasmid, and replacement-specific primer combinations are indicated by arrows and expected sizes of diagnostic PCRs are given. *B.* Genotyping indicates transient replacement parasites (left panel). Lane 1: PCR of 3′-replacement fragment using primer pair b3D-1 and Pb-11 which amplified a PCR fragment of 1.18 kb. The fragment was subcloned and its nucleotide sequence verified. Lane 2: PCR using b3D-1 and b3D-2 to amplify a 1.15 kb fragment diagnostic for the recombined episome. Lane 3: PCR diagnostic for presence of wild-type parasites using primer combination Pb-3 and Pb-12 amplifying a 1.84 kb fragment which was sequenced to verify its authenticity. *C.* Diagnostic PCRs after the mixed population was transferred into a fresh animal. Lane 1: PCR with primer pair Pb-10 and b3D-3 to amplify the 5′-integration fragment of 1.02 kb. No clear product was detectable. Lane 2: PCR with primer pair b3D-1 and Pb-11 to amplify the 3′-integration fragment of 1.18 kb. No product was detectable. Lane 3: PCR of episomal recombined plasmid using b3D-1 and b3D-2 to amplify a 1.15 kb band. The fragment was subcloned and its sequence verified. Lane 4: PCR diagnostic for presence of wild-type parasites using primer combination Pb-10 and Pb-11 amplifying a 2.47 kb fragment which was sequenced to verify its authenticity.

The second approach in the *P. berghei* system was to replace the endogenous *PbLplA1* gene with the selectable marker *T. gondii DHFR-TS* using the transfection plasmid b3D-*LplA*-REP. Linearised DNA was transfected at 6 independent occasions and pyrimethamine resistant parasites were analysed by PCR (Fig. 8). The results showed that re-circularisation of the linearised plasmid had occurred in 5 out of the 6 occasions, which usually leads to loss of linearised DNA because the drug pressure applied selects for the presence of the circular plasmid containing the selectable marker. In those parasites, the gene locus was still intact as expected. However, in parasite line 6, it was possible to amplify the diagnostic product for the *PbLplA1* gene replacement after two rounds of PCR (Fig. 8B). Again this was verified by subcloning the PCR fragment and analysing its nucleotide sequence. However, this mutant parasite line was lost after transfer into a new animal - a procedure that is routinely performed to propagate the knockout parasite population and to be able to generate clones (Fig. 8C). These data support that the *LplA1* locus can be targeted not only by the control plasmid, but also by the replacement plasmid. However, the results suggest that the knockout of the *LplA1* gene might have severe effects on parasite growth rate and survival during erythrocytic development.

## Discussion

The ligation of LA to apo-E2 subunits of the KADH or the apo-H-protein of the GCS is likely to be an essential reaction in most organisms, unless it can be replaced by the LA biosynthesis pathway like in most bacteria [6]. In *Plasmodium* LA biosynthesis and salvage are located in two different organelles, the mitochondrion and the apicoplast [8], [10]–[12]. It further appears that LA cannot be exchanged between the two organelles suggesting that knockout of the mitochondrial salvage pathway might be lethal for the parasites [13]–[15]. However, *Plasmodium* possesses two LplA-like proteins with LplA1 present in the mitochondrion and LplA2 present in both mitochondrion and apicoplast [11], [12], [16]. This situation raises the question whether there is redundancy between the two ligases as it was shown that LplA2 can partially compensate for LipB function in the apicoplast [16]. However, our data do not give conclusive information about potential redundancy between LplA1 and LplA2 in *P. falciparum*, because it was impossible to target the *LplA1* gene locus, even in the presence of an expressing episomal copy of the gene; neither was it possible to target the *P. falciparum LplA1* locus with a knock-in construct (see Fig. 2 and 4).

Therefore, we decided to expand our efforts and attempt a knockout of the *LplA1* gene in the murine malaria parasite *P. berghei*. The *P. berghei LplA1* gene locus is targetable by knock-in and also a gene replacement construct, clearly demonstrating that this gene locus is not refractory to recombination in *P. berghei*. Given that the genetic organisation of the *LplA1* gene locus is highly syntenic in *P. falciparum* and *P. berghei*, it is possible that the failure to obtain a gene disruption of *LplA1* in *P. falciparum* is due to its important function for the parasites during their erythrocytic development. This was also suggested by Allary and colleagues [13] who showed that inhibition of *P. falciparum* LplA1 by the LA analogue 8-bromooctanoic acid had deleterious effects on parasite survival. Thus, it appears that LplA1 function cannot be replaced by LplA2 despite the dual targeting of this protein into apicoplast and mitochondrion. One reason for this lack of redundancy might be the different expression profiles of *LplA1* and *LplA2* genes in *P. falciparum*; *PfLplA1* mRNA is primarily present throughout the intraerythrocytic life of the parasites while *PfLplA2* seems to be primarily expressed in the sexual stages of the parasites [22]. Another possible explanation for the lack of redundancy between the two proteins might be their differential substrate specificities as has previously been suggested by Allary et al. [13]. This is not unusual and has been described before in *Listeria monocytogenes*, where the two LplAs present in the LA auxotroph organism act on distinct substrates [23], [24]. Thus, substrate specificity and time of expression might regulate the function of *LplA1* and *LplA2* in *P. falciparum*. Our preliminary data on the effect of a *LplA2* knockout in *P. berghei* suggest, however, that the protein is not essential for blood stage development and has a function important for the parasites' development in the insect host, which is consistent with the expression profile of this gene (Günther, Matuschweski and Müller, unpublished data).

The *P. berghei LplA1* gene locus was not only targeted by a knock-in construct but also by the gene replacement construct, which exchanges the *PbLplA1* gene with the selectable marker and consequently deletes the *PbLplA1* gene. However, it was impossible to characterise the *PbLplA1^(−)^* mutants because the initial population of *P. berghei LplA1* null-mutants that was obtained appeared to be out-competed by wild-type parasites carrying an episomal selectable marker. This suggested that the development/growth of *LplA1* null-mutants was severely compromised implying an important role for *P. berghei* during blood stage development.

In conclusion our data suggest that the *LplA1* gene in *P. falciparum* is refractory to recombination, which excludes conclusive information as to whether the gene is essential for the survival of the asexual stages of this human pathogenic *Plasmodium* species *in vitro*. Two complementary strategies to select *LplA1* loss-of-function mutant parasites of *P. berghei* were employed. Consistent with a severe defect in a null-mutant we could not obtain viable *LplA1^(−)^* parasite clones, indicating that *LplA1* has a crucial function in *Plasmodium* during their intraerythrocytic life and that development of LplA1 inhibitors has potential for future drug discovery.

## Materials and Methods

### Materials

Plasmids pHH1, pHBG and pHrBI-1/2 were kind gifts of Professor A.F. Cowman (The Walter and Eliza Hall Institute, Melbourne, Australia) and Professor G.I. McFadden (University of Melbourne, Melbourne, Australia), respectively. Plasmid b3D.DT∧H.∧D was a kind gift of Professor A. P. Waters (Leiden University, The Netherlands) [25]. *P. berghei* was grown in NMRI (Naval Medical Research Institute) outbred mice (Charles River Laboratory, Sulzfeld, Germany), or Sprague-Dawley outbred rats (Charles River Laboratory, Sulzfeld, Germany).

### Parasites

*P. falciparum* D10 (Papua New Guinea) were cultured according to [26] in complete RPMI 1640 medium containing 0.1% Albumax II (Invitrogen) at 5% haematocrit. The cultures were maintained at 37°C in an atmosphere of reduced oxygen (3% CO_2_, 1% O_2_, 96% N_2_). Before transfection with pHH1-*LplA1*-KO, pHH1-*LplA1*-KOkon or pHrBI-*PbLplA1*, parasites were synchronised using sorbitol according to [27]. Genomic DNA of parasites was isolated using the QIAamp DNA Mini Kit (Qiagen) after parasites were isolated from the red blood cells through saponin lysis [28]. Chromosome blocks for pulse field gel electrophoresis were generated according to standard procedures. Briefly, a 10 ml culture with approximately 5% parasitemia was saponin-lysed and the parasites were pelleted by centrifugation. The parasite pellet was resuspended in 3 pellet volumes of warm (∼50°C) phosphate buffered saline (PBS) and the same volume of 2% (w/v) warm low melting point agarose (Invitrogen) was added and the mixture, transferred to plug-molds (BioRad) and allowed to set. Subsequently, the blocks were transferred into 10 mM Tris/HCl buffer pH 8.0 containing 0.5 mM EDTA, 1% (v/v) sarkosyl and fresh proteinase K at 2 mg/ml and incubated for 48 h at 37°C to free the nucleic acids. Chromosome blocks were stored for up to 7 months in 10 mM Tris/HCl buffer pH 8.0 containing 50 mM EDTA at 4°C.

Protein extracts were prepared from saponin-isolated parasites by resuspending the pellets in lysis buffer (100 mM HEPES (pH 7.4), 5 mM MgCl_2_, 10 mM EDTA, 0.5% (v/v) TritonX-100, 5 µg/ml RNAse, 1 mM phenylmethylsulphonyl fluoride, 1 mM benzamidine, 2 µg/ml leupeptin, 10 µM E-64, 2 µM 1,10-phenanthroline, 4 µM pepstatin A) followed by three cycles of freeze/thawing and sonication in a sonicating water-bath (Fisherbrand). Protein concentrations were determined using the Bradford assay [29].

Asynchronous blood-stages of *P. berghei* (strain NK65) were maintained in NMRI mice. For isolation of synchronized late stage schizonts 5 ml infected blood was obtained by cardiac puncture from a Sprague/Dawley rat and cultured overnight in RPMI 1640, supplemented with 20% (v/v) fetal calf serum, 25 mM HEPES, 25 mM glutamine, and 14 mg/ml gentamycin. Parasites were isolated by gradient centrifugation and used for subsequent transfection with the constructs b3D-*LplA1*-int1 and int2 and b3D-*LplA1*-REP. Genomic DNA of *P. berghei* parasites was isolated from blood obtained from an infected animal by cardiac puncture and erythrocytes, lymphocytes and platelets were separated using a cellulose column. The red blood cells were then lysed in 0.2% (w/v) saponin in PBS and after washing the resulting parasite pellet in PBS, it was resuspended in 200 µl of PBS and stored at −20°C before genomic DNA was extracted using the QIAamp DNA Mini Kit (Qiagen).

### Transfection of parasites

The constructs pHH1-*LplA1*-KO and pHH1-*LplA1*-KOkon were generated using the oligonucleotides Pf-1/Pf-2 and Pf-3/Pf-4, respectively (see Table 1). The primers introduced 5′ of the PCR products a *Bgl*II site and 3′ a *Xho*I site that allowed directional cloning of the products into pHH1 [30]. PCR was performed with *Pfu* polymerase and genomic *P. falciparum* DNA. The knockout construct is truncated at its 5′ end lacking the ATG start codon and at the 3′ end, a premature stop codon was introduced 237 bp upstream of the natural stop codon. This would result in the formation of two incomplete and inactive *LplA1* copies upon single cross-over recombination of the plasmid in the *LplA1* gene locus. In contrast, the control construct, corresponding to nucleotides 206–1227, retains the endogenous C-terminus of the *LplA1* gene but lack the 5′ end and thus should generate a functional copy of *LplA1* and a non-functional pseudogene upon recombination. It will, however, introduce an artificial 3′ UTR downstream of the first *LplA1* copy [30]. The expression plasmid pHrBI-*PbLplA1* encompasses the entire ORF of the *P. berghei LplA1* gene, which was amplified from *P. berghei* genomic DNA using the oligonucleotides Pb-1 and Pb-2 (see Table 1). The introduced restriction sites *Bgl*II and *Not*I allowed directional cloning of the PCR product into the Gateway entry vector pHGB [31]. Through recombination using the single site Gateway cloning system (Invitrogen), the *PbLplA1* ORF was transferred into the destination vector pHrBI-1/2 which contains the blasticidin S deaminase (BSD) as a selectable marker [31]. All PCR products were initially subcloned into TOPO-Blunt (Invitrogen) and their sequences were verified (The Sequencing Service, University of Dundee, UK, www.dnaseq.co.uk) before cloning into the transfection plasmid pHH1 or entry vector pHGB. Transfection of the constructs was carried out as described before [32], [33] and parasites were selected with either 5 nM WR99210 or 2 µg/ml blasticidin (Invitrogen). The expression plasmid pHrBI-*PbLplA1* was transfected into *P. falciparum* D10 and the *P. falciparum* line carrying the pHH1-*LplA1*-KO plasmid (cycle 0).

**Table 1:** Oligonucleotides used in this study.

Primer name	Sequence	Restriction site
Pf-1	5′-GCGC**AGATCT**AAACGAATATTCAGGTTGG-3′	BglII
Pf-2	5′-GCGC**CTCGAG**CTACCAAATATGATTTTGAAATTTGGG-3′	XhoI
Pf-3	5′-GCGC**AGATCT**GGAGAAATAACCGATCTATAATTATAGG-3′	BglII
Pf-4	5′-GCGC**CTCGAG**CTAAAGTTCTTGTAATATCCATGAACG-3′	XhoI
Pb-1	5′-GCGC**AGATCT**ATGCCAGGTATATCCTGTTTTGTAAAACGATGTTATGG-3′	BglII
Pb-2	5′-GCGC**GCGGCCGC**TTAGAGTTCTTCTAATATCCATGAAGTTATTTCTTCTAAGG-3′	NotI
Pb-3	5′-GCGC**GGATCC**CAAAATATTTACTTTAATTTATCGTTGG-3′	BamHI
Pb-4	5′-GCGC**CCGCGG**TTAGTCTAATGCATCTGAAAAAACATTTCC-3′	SacII
Pb-5	5′-GCGC**CCGCGG**GGACAAGCATAGCTTATGCCCGATC-3′	SacII
Pb-6	5′-GCGC**GGTACC**TACATTATATTTAATATATAACAGGG-3′	KpnI
Pb-7	5′-GCGC**AAGCTT**CCAACGATAAATTAAAGTAAATATTTTG-3′	HindIII
Pb-8	5′-GCGC**GCGGCCGC**CCAATACTTTAAAACATTTAACAATC-3′	NotI
Pb-9	5′-GCGC**CCGCGG**GGACAAGCATAGCTTATGCCCGATC-3′	SacII
Pb-10	5′-GGATAATGTAATAAAATCTAGCCATTTAACTC-3′	
Pb-11	5′-GCGCGTGTTGGTGTGTATATGAGAAATTCC-3′	
Pb-12	5′-GTGTTGGTGTGTATATGAGAAATTCC-3′	
b3D-1	5′-CCCGCACGGACGAATCCAGATGG-3′	
b3D-2	5′-GCGCCGACGTTGTAAAACGACGGCC-3′	
b3D-3	5′-CGCATTATATGAGTTCATTTTACACAATCC-3′	

The b3D.DT∧H.∧D-based knockout construct [25] for single homologous recombination in *P. berghei* encompasses a 1014 bp *PbLplA1* fragment corresponding to nucleotides 88 to 1098 (lacking the last 147 bp) which was amplified using the oligonucleotides Pb-3 and Pb-4 (see Table 1), *Pfu* polymerase and genomic *P. berghei* DNA. An artificial stop codon was introduced at the 3′ end of the PCR product and the fragment was cloned into the *BamH*I and *Sac*II sites of one of the multiple cloning sites of the vector resulting in b3D-*LplA1*-int1. Similar to the strategy described above for *P. falciparum*, transfection with this construct should result in the disruption of the gene locus and the formation of two incomplete and inactive copies of *LplA1*. The control plasmid b3D-*LplA1*-int2 encompasses a 1734 bp PCR fragment and was amplified using Pb-3 and Pb-5. As the knockout construct, it was cloned into the *BamH*I and *Sac*II sites of b3D.DT∧H.∧D. This fragment is truncated at the N-terminus of the gene (lacking the first 87 bp) but comprises the full–length C-terminus including the endogenous stop codon and 3′UTR and single homologous recombination should therefore result in the reconstitution of the endogenous gene. Both plasmids were digested with *Hpa*I to linearise the vector before transfection into NK65 wild-type parasites according to [25]. The transfections were performed one to three times. In a second strategy PbLplA1 5′ UTR and 3′ UTR regions were cloned into the two multiple cloning sites of b3D.DT∧H.∧D using the oligonucleotides Pb-6/Pb-7 and Pb-8/Pb-9, respectively (see Table 1). The 5′ UTR PCR fragment was 480 bp in length and contained *Kpn*I and *Hind*III restriction sites; the 3′UTR PCR fragment was 516 bp long and had *Not*I and *Sac*II restriction sites that allowed directional cloning. The resulting plasmid was called b3D-LplA1-REP. Using *Kpn*I and *Sac*II, the transfection cassette (containing the two *LplA1* UTRs and the selectable marker *T. gondii* dihydrofolate reductase-thymidylate synthase (*TgDHFR-TS*) was isolated from the plasmid backbone, and digested plasmid was transfected according to [25]. The transfection was repeated at 6 independent occasions.

### Southern blot analyses

For each *P. falciparum* line that was analysed, 1 to 5 µg of genomic DNA was digested with *Nde*I, separated on a 0.8% (w/v) agarose gel and blotted onto positively charged nylon membrane (GE Healthcare) using standard methods [34]. The blots were prehybridised in 5× SSC, 0.1% (w/v) SDS, 5% (w/v) dextrane sulphate and 1∶20 dilution of GI liquid block provided with the Gene Images CDP-Star detection kit from GE Healthcare. After 2 h at 60°C either the fluoresceine-labelled *LplA* coding region, the *human DHFR* (*hDHFR*) or the *blasticidin S deaminase* (*BSD*) probes, generated using the Gene Images Random Prime labelling module according to the manufacturer's recommendation (GE Healthcare), were added and the blots hybridised overnight at the same temperature. After washes with decreasing salt-concentrations, the signals were detected by incubating the blots with an anti-fluoresceine alkaline phosphatase conjugated antibody followed by several washes in 100 mM Tris HCl pH 9.5 containing 300 mM NaCl and 0.3% (v/v) Tween 20. After application of detection solution the blot was exposed to autoradiography film (Kodak).

### Pulsed field gel electrophoreses

To separate *P. falciparum* chromosomes on an agarose gel, pulse field gel electrophoreses were performed using the CHEF-DR III Variable Angle System (BioRad). The conditions used in this study are optimal to separate chromosomes 11 to 14 because the *LplA1* gene is located on chromosome 13. The gel consisted of 1% (w/v) agarose in 1× TAE and the separation was performed using the following parameters: 360–800 s pulse, 3 V/cm^2^ (100 Volts) for 96 hours. The gels were blotted and probed as described above.

### Western blot analyses

To detect whether *P. falciparum* transfected with pHrBI-*PbLplA1* alone or *P. falciparum* co-transfected with pHH1-*LplA1*-KO and pHrBI-*PbLplA1* expressed *P. berghei* LpA1 protein in addition to the endogenous *P. falciparum* LplA1 protein, parasite extracts of wild-type and mutant parasites were subjected to western blot analyses. Briefly, 15 µg of *P. falciparum* lysates or the protein extract obtained from 1×10^6^ or 0.2×10^7^ *P. berghei* were separated on a 4–12% SDS-PAGE (Invitrogen) and then blotted onto nitrocellulose (Schleicher and Schüll), using standard techniques [34]. The blots were incubated with a rabbit anti-LplA1 antibody (generated against recombinant protein *P. falciparum* LplA1 by Eurogentec, Belgium) at a dilution of 1∶5000 and the secondary anti-rabbit IgG (H+L), HRP conjugate (Promega) at a dilution of 1∶10,000 before being developed using the Immobilon™ Western Chemiluminescent HRP Substrate (Millipore).

### PCR analyses of *P. berghei* transfectants

The parasite populations that were isolated from infected mice after transfection of the three *P. berghei* constructs were analysed by PCRs using diagnostic primer combinations that allowed determining whether (i) the gene locus had been targeted by the plasmid, (ii) the endogenous gene was still present and (iii) the plasmid had recombined and was present as an episome. The parasites that were transfected with b3D-LplA1-int1 were analysed with primer pair Pb-10/Pb-11 which should result in the amplification of a 2479 bp fragment if the endogenous gene was still present. In order to analyse whether the recombined transfection plasmid was present in the parasite population, primers b3D-1 and b3D-2 were used, amplifying a 1628 bp fragment. For amplification of PCR products diagnostic for the homologous recombination of the transfected construct with the gene locus, the two primer sets b3D-1/Pb-11 and Pb-10/b3D-2 were used to analyse the 3′ and 5′ integration-specific events, respectively. The parasites transfected with the control plasmid b3D-LplA1-int2 were analysed with the same primer sets.

Parasites transfected with the replacement construct b3D-LplA1-REP were analysed using the gene locus specific primer set Pb-3/Pb-11 (amplifying a 1837 bp product) or Pb-10/Pb-12 (amplifying a 2479 bp product). These parasite lines were also analysed for the presence of plasmid using the primer pair b3D-1 and b3D-2, amplifying a 1150 bp fragment. Diagnostic PCRs for the integration event were performed for the 3′ and 5′ integration site using the primer combinations b3D-1/Pb-11 (1183 bp) and Pb-10/b3D-3 (1015 bp), respectively (for primer sequences see Table 1).
